# Knowledge, Attitudes, and Practices about Electronic Personal Health Records: A Cross-Sectional Study in a Region of Northern Italy

**DOI:** 10.1007/s10916-024-02065-z

**Published:** 2024-04-17

**Authors:** Giacomo Scaioli, Manuela Martella, Giuseppina Lo Moro, Alessandro Prinzivalli, Laura Guastavigna, Alessandro Scacchi, Andreea Mihaela Butnaru, Fabrizio Bert, Roberta Siliquini

**Affiliations:** 1 Department of Public Health Sciences and Paediatrics, University of Turin, Via Santena 5bis, Turin, 10126 Italy; 2 Infection Control Unit, ASL TO3, Turin, Italy; 3 AOU City of Health and Science of Turin, Turin, Italy

**Keywords:** Electronic personal health records, Health data, e-health, Telemedicine, Patient-centered care

## Abstract

The Electronic Personal Health Record (EPHR) provides an innovative service for citizens and professionals to manage health data, promoting patient-centred care. It enhances communication between patients and physicians and improves accessibility to documents for remote medical information management. The study aims to assess the prevalence of awareness and acceptance of the EPHR in northern Italy and define determinants and barriers to its implementation. In 2022, a region-wide cross-sectional study was carried out through a paper-based and online survey shared among adult citizens. Univariable and multivariable regression models analysed the association between the outcome variables (knowledge and attitudes toward the EPHR) and selected independent variables. Overall, 1634 people were surveyed, and two-thirds were aware of the EPHR. Among those unaware of the EPHR, a high prevalence of specific socio-demographic groups, such as foreign-born individuals and those with lower educational levels, was highlighted. Multivariable regression models showed a positive association between being aware of the EPHR and educational level, health literacy, and perceived poor health status, whereas age was negatively associated. A higher knowledge of the EPHR was associated with a higher attitude towards the EPHR. The current analysis confirms a lack of awareness regarding the existence of the EPHR, especially among certain disadvantaged demographic groups. This should serve as a driving force for a powerful campaign tailored to specific categories of citizens for enhancing knowledge and usage of the EPHR. Involving professionals in promoting this tool is crucial for helping patients and managing health data.

**Supplementary Information:**

The online version contains supplementary material available at 10.1007/s10916-024-02065-z.

## Introduction

The Electronic Personal Health Record (EPHR) is an innovative repository for storing personal health data managed by the citizens [1, 2]. It has been implemented in several countries to promote patient-centred care, self-management of chronic illness, and communication with healthcare professionals [3–5]. The EPHRs enhance patient safety by preventing diagnostic or medication errors and recording updated prescribed treatment plans, over-the-counter supplements or medications, and diagnostic test results. Furthermore, the EPHRs potentially reduce geographical barriers, especially in fragmented healthcare systems, improving continuity of care and efficiency, even among patients with comorbidities and severe mental illnesses [6, 7].

Positive outcomes of EPHRs were observed in the follow-up of cancer patients and in the management of other chronic diseases, such as diabetes, achieving better clinical outcomes (e.g., improvedhaemoglobin A1c levels, fewer hospitalisations, and emergency room visits) [7, 8]. The EPHRs could positively impact patients with multiple sclerosis, breast cancer, or more rare conditions [9–11].

Moreover, EPHRs offer several functionalities for healthcare providers, supporting them in organising reliable health information and appropriate assistance for prompt monitoring and responding to patients’ requests [12, 13].

Nevertheless, the utilisation of the EPHR among the community and health professionals still needs to be improved [14]. The “EPHR paradox” refers to a significantly low overall adoption of this tool, despite high consumer acceptance and expected benefits [15]. Among threats and barriers to adopting the EPHR, individual factors such as old age, low socioeconomic status, low educational level, and good health status negatively influence the attitudes toward the EPHR [3, 16, 17]. Organisational and technical factors, inadequate interoperability and usability of the system, low perception of security, privacy, and data quality, and the inadequate customisation of the EPHR contribute to its underutilisation [13].

Overall, an advanced evaluation of citizens’ opinions and intentions about the EPHR is still lacking in many countries [18–22].

The implementation of the EPHR worldwide has developed unevenly in different countries, including EU countries. [23] England created a health information infrastructure in the National Health System (NHS) through the ‘NHS app’ [24]. In Denmark, a portal called ‘sundhed.dk’ was created in 2004 [25]. In France, ‘My Health Space’ has been active since 2022 [26]. In Germany, a digital portal called ‘Elektronische Patientenakte’ or ‘ePA’ was implemented [27].

Attempts to level out the medical information across Europe have been made through the eHealth Digital Service Infrastructure (eHDSI), which was created to ensure the continuity of care for European citizens while they are travelling abroad in the EU, giving EU countries the possibility to exchange health data in a secure, efficient and interoperable way [28]. In Italy, the availability of the EPHR dates back to 2007. Nonetheless, its implementation became mandatory in 2012.

The EPHR in Italy consists of two main components: the ‘minimum core’, which is the same in all Italian regions, and the ‘additional data and documents’ part, which differs according to the choices and level of maturity of the digitisation process in each region. The main functions and information a patient can find on his/her EPHR are patient summary, patient’s personal notebook, prescriptions, appointments (outpatient visits, hospitalisations, etc.), medical records, health reports, diagnostic and therapeutic plans, residential and semi-residential care, drugs, vaccinations, specialist, emergency, and surgical services, medical certificates [29, 30]. Physicians, prior authorisation, have access to the same information. Patients might also add their documents released from both public and private healthcare facilities [29].

Currently, the EPHR in Italy is different in each Italian region; each region has its own autonomy in the choices regarding the modality of implementation of the EPHR, leading to a potential disequilibrium in the usability of the platform among the different Regions. This reflects also on the activation rate of the EPHRs in the Italian regions, which ranges between 85% and 100%. Moreover, underutilisation is reported in almost all regions [30]. The reasons for this underutilization are still unknown, given that previous research only focussed on the technical features of the implementation of the EPHR. Thus, this is the first Italian study to investigate the citizens’ opinions about the usefulness and potentialities of the EPHR [31], with the aim to assess knowledge and attitudes about the EPHR, and to define determinants and barriers to the diffusion of the EPHR. The main objectives are to determine the awareness, level of knowledge, and attitudes about the EPHR, its implementation among the general population the identification of specific barriers to its use and potential predictors (socio-demographic factors, citizens’ health status, and literacy-related factors) of knowledge and attitudes toward the EPHR.

## Methods

A region-wide cross-sectional study was carried out between March and December 2022. A paper-based and online survey was shared among people currently residing in Piedmont, a northern Italy region where only 13% of the citizens use the EPHR [24]. The target population was adults from the age of 18 onwards.

Informed consent was required to access the questionnaire, and participation was voluntary, anonymous, and without compensation. A brief introduction informed the participants about the aims and objectives of the research. All the procedures followed the 1964 Helsinki Declaration and its later amendments [32]. The Ethics Committee of the University of Turin approved the study protocol (nr. 0169989, dated 14.03.2022).

The paper-based survey was distributed in outpatient clinics and vaccination settings, whereas the web-based format was shared on social media platforms (mainly Facebook, Twitter, and WhatsApp).

After a preliminary literature review, the research group elaborated on the survey, shaped by the Knowledge, Attitudes, and Practices (KAP) model [33].

Overall, four different sections made up the questionnaire. The first general part contained items concerning the socio-demographic characteristics of the sample. The following three sections comprised questions regarding health status, literacy, experience as users of digital health applications, and knowledge, attitudes, and practice about the EPHR. Specific questions of these sections investigated the modality of access, management, and usefulness of the EPHR.

### Statistical Analysis

The Shapiro-Wilk normality test was performed to assess normally distributed variables. Descriptive analyses were reported with frequency and percentages for categorical variables and mean and standard deviation (SD) for continuous ones. Missing data were excluded.

The primary outcomes were: awareness of the EPHR (assessed by asking: “Have you ever heard about the EPHR?”) as a binary outcome variable, and scores on knowledge and attitudes about the EPHR. Specifically, the knowledge score (KS) about the EPHR was assessed through nine questions about its availability, accessibility, functions, and usability. A score from 0 to + 9 was computed by ascribing one point to each correct answer.

Attitudes about the EPHR were assessed through five “four-level” Likert items (ranging from one - strongly disagree, to four – strongly agree) on its usefulness and impact on health status and healthcare assistance, and the availability of personal data for health professionals within the EPHR. A minimum of one point to a maximum of four points were attributable to each question, scoring overall from + 5 to + 20 (Attitudes-score, AS). A higher score corresponds to more positive attitudes toward the EPHR.

Univariable and multivariable logistic regression models analysed associations between multiple independent variables and the dichotomous outcome variable “Have you ever heard about the EPHR?“. Independent variables were socio-demographic variables (such as gender, age, country of origin, the size of the place in which they live, education, family/housing unit, profession), health-related information (as health status, being affected by chronic disease(s), frequent request of medical assistance, self-confidence in filling out medical papers, gathering all medical reports before GP/specialist’s visit, trust in GPs and medical specialists), and also having filled the questionnaire in paper form or electronically.

Univariable and multivariable linear regression analyses investigated the association between continuous outcome variables (KS and AS) and the above mentioned independent variables.

The selection of the variables was a backward stepwise selection, with *p* < 0.25 as a cut-off [34].

Results were expressed as adjusted Odds Ratio (adj OR) for logistic regressions and adjusted coefficients (adj Coeff) for linear regressions, and 95% Confidence Interval (CI). Statistical significance was set at *p* < 0.05.

All analyses were performed using the Stata Software 17.0 (Stata Corp, College Station, Texas 77,845 USA).

## Results

### Descriptive Analysis

Overall, 1634 people were surveyed. Almost two-thirds were female, the mean age was 48 years (SD ± 13.8), and 95.6% were Italian. Most participants declared to live in a small- or medium-sized town (below 30 thousand inhabitants). Educational level was mainly “high school” or “college degree” (50.4% and 25.8%, respectively), and most participants stated to be employed (69.3%). Health Care Workers (HCWs), primarily nurses, comprised less than 20% of the population (Table 1).

**Table 1:** Socio-demographic characteristics of the sample

Variable		n (1634)	%
Gender	Female	1,214	74.30%
Age	Mean and standard deviation	47,93	± 13.79
Nationality	Italian	1,562	95.59%
Place of residence	≤ 5,000 habitants	764	46.79%
	5,001–30,000 habitants	549	33.62%
	30,001–50,000 habitants	122	7.47%
	50,001–100,000 habitants	93	5.70%
	100,001–250,000 habitants	14	0.85%
	> 250,000 habitants	91	5.57%
Study title	Middle school or lower	269	16.47%
	High School	823	50.37%
	College degree or higher	542	33.16%
Profession	Student	54	3.30%
	Employed	1.132	69.28%
	Housekeeper	105	6.42%
	Retired	250	15.30%
	Unemployed	63	3.86%
	Other	30	1.84%
Healthcare worker	Yes	318	19.47%
Lives alone	Yes	190	12.02%

Most respondents declared an excellent/good health status (75.4%) and no chronic pathologies (50.3%) without requiring medical assistance in the last three months. Respondents were mostly “Quite a bit” confident in filling out medical forms independently (Table 2).

**Table 2:** Health-related characteristics of the sample

Variable		N (1634)	%
How do you consider your health status?	Excellent	307	19.46%
	Good	883	55.96%
	Fair	354	22.43%
	Bad	34	2.15%
Do you have any chronic illnesses?	Yes	814	49.73%
How often have you been visited by a doctor / hospitalized in the last three months?	Never	897	56.84%
	Once	383	24.27%
	Twice	143	9.06%
	Three times	63	3.99%
	More than three times	92	5.83%
How often do you have someone helping you read medical materials (such as reports. discharge letters)?	Always	62	3.94%
	Often	95	6.03%
	Sometimes	191	12.13%
	Rarely	311	19.75%
	Never	916	58.16%
How confident do you feel in filling out medical forms on your own?	Very much	290	18.37%
	A lot	512	32.43%
	Quite a bit	620	39.27%
	A little	129	8.17%
	Not at all	28	1.77%
When you visit a doctor or specialist. do you bring documents concerning your health condition?	Always	905	57.35%
	Often	351	22.25%
	Rarely	227	14.39%
	Never	95	6.01%
Do you use a computer or smartphone to manage your health?	Yes	1.632	99.45%
Do you believe that your General Practitioner (Family Doctor) has the necessary information about your health?	Yes complete information	429	27.66%
	Yes, but not complete	753	48.55%
	Yes, a minority of the information	228	14.70%
	Very few/no information	141	9.09%
Do you believe that the specialist doctors who have examined you in the past had the necessary information about your health?	Yes, complete information	165	10.56%
	Yes, but not complete	534	34.17%
	Yes, a minority of the information	357	22.84%
	Very few/no information	471	30.13%
	A Specialist has never examined me	36	2.30%

A total of 965 participants (61.3%) were aware of the EPHR (Table 3). People who heard of the EPHR were primarily women, Italians, highly educated, employed (especially Heath Care Workers, HCWs), not living alone, highly self-confident in filling medical reports, with chronic conditions, and frequent users of healthcare services (Table S3 and S4).

**Table 3:** Multivariable logistic regression analysis. Outcome: being aware of the EPHR. All the variables of the table were included in the multivariable model

Variabile		OR*	95% CI**
Gender	Female	0.86	0.65–1.14
Age		0.99	0.98–1.01
Nationality	Italian	Ref	Ref
	Foreigner	**0.37**	**0.20–0.68**
Place of residence	≤ 5,000 inhabitants	Ref	Ref
	5,001–30,000 inhabitants	0.91	0.70–1.18
	30,001–50,000 inhabitants	1.05	0.63–1.75
	> 50,000 inhabitants	**0.65**	**0.44–0.96**
Lives alone	Yes	**0.59**	**0.41–0.83**
Study title	Middle school or lower	Ref	Ref
	High School	**1.66**	**1.19–2.32**
	College degree or higher	**2.01**	**1.38–9.94**
Profession	Student/employer	Ref	Ref
	Housekeeper/retired/ unemployed	**0.68**	**0.50–0.94**
Health care worker	Yes	**1.65**	**1.18–2.32**
How do you consider your health status?	Excellent/good	Ref	Ref
	Fair/poor	**1.69**	**1.26–2.26**
How often have you been visited by a doctor / hospitalized in the last three months?	Never	Ref	Ref
	At least once	1.214	0.955–1.544
How confident do you feel in filling out medical forms on your own?	Very much	Ref	Ref
	A lot	0.65	0.45–0.93
	Quite a bit	**0.39**	**0.27–0.55**
	A little	**0.39**	**0.23–0.64**
	Not at all	**0.07**	**0.02–0.27**
When you visit a doctor or specialist, do you bring documents concerning your health condition?	Always	Ref	Ref
	Often	**0.80**	**0.60–1.06**
	Rarely	**0.63**	**0.44–0.88**
	Never	**0.43**	**0.26–0.71**
Do you believe that your General Practitioner (Family Doctor) has the necessary information about your health?	Yes, complete information	Ref	Ref
	Yes, but not complete	0.88	0.66–1.16
	Yes, a minority of the information	0.91	0.62–1.33
	Very few/no information	**0.55**	**0.35–0.87**
Filled the questionnaire in paper	Yes	**0.26**	**0.17–0.39**

Abbreviation:

* OR = Odds Ratio; ** 95% CI = 95% Confidence Interval; Ref: Reference variable

*p*-value < 0.05 *(bold numbers are significant results)*

The mean KS was 6.0, SD ± 1.1. Regarding the AS, the mean result was 16.6 (SD ± 2.9).

### Multivariable Regression Models

Multivariable regression model of the binary outcome “being aware of the EPHR” showed that foreign people (adj OR 0.36, *p* = 0.001), as well as people living in towns with more than 50 thousand inhabitants (adj OR 0.64, *p* = 0.030) were less likely to be aware of the EPHR.

Educational level as “high school” or “college/postgraduate degree” was significantly associated with being aware of the EPHR (adj OR 1.66, *p* = 0.003 and adj OR 2.01, *p* < 0.001) compared to persons with a lower educational level. People not employed were less likely, and HCWs were more likely, to be aware of the EPHR (adj OR 0.68, *p* = 0.018; adj OR 1.65, *p* = 0.004). (Table 3)

Participants who declared a fair/poor health status were more aware of the EPHR than healthy people (adj OR 1.69, *p* < 0.001). Also, self-confidence in filling out medical reports was positively associated with having heard of the EPHR (Table 3). Believing that the GP has limited information about the respondent’s health leads to a significant reduction in the probability of being aware of the EPHR, compared to believing that the GP has access to all the information about the respondent’s health (adj OR 0.55, *p* = 0.010). Lastly, having filled out the paper questionnaire gave a lower chance of being aware of the EPHR than having filled out the electronic questionnaire (adj OR 0.26, *p* < 0.001) (Table 3).

Linear regression models regarding the KS highlighted that age was negatively associated with a high score (adj Coeff − 0.01, *p* = 0.004). Moreover, the availability of support in comprehension of medical documents/reports related to better knowledge (Table supplementary material 5 S5).

Concerning the AS, the multivariable linear regression model showed that elderly people seemed to be less inclined to accept the EPHR (adj Coeff − 0.03, *p* < 0.001)(table supplementary material 6 S6). A higher KS was associated with a higher AS (adj Coeff 0.27, *p* = 0.003).

## Discussion

The study aimed to assess the prevalence of awareness and acceptance of the EPHR in northern Italy and to define determinants and barriers to its implementation. About 40% of the sample was unaware of the EPHR, whereas one-third did not access it within the last year, despite being aware of its existence. According to recent data, only 13% of the inhabitants of the Piedmont Region accessed their own EPHR in the previous 90 days [35].

As confirmed by previous findings, higher educational level was associated with a more heightened awareness of the EPHR [36]. According to the results of the present paper, confidence in filling out medical forms is related to a lower likelihood of awareness of the EPHR, suggesting that health literacy predicts awareness on this topic. However, the educational level and the health literacy level alone are not sufficient to explain the level of use of the EPHR, since the EPHR “might be difficult even for patients with relatively high levels of education, if they do not also have experience in using the Internet” (digital literacy) [36]. Previous research from the UK showed lower awareness of the EPHR among foreign individuals, lower-education qualified people, and people with low digital literacy [37].

In the present study, individuals who completed the paper-based questionnaire are less likely to be aware of the EPHR than those who completed the online format, after adjusting for age, gender, socio-demographic, and health literacy-related variables. This might be explained by factors linked to digital literacy, that might influence awareness regarding the EPHR. In this regard, the higher online participation explains the excellent level of knowledge of the EPHR, compared to the general population. This is consistent with findings in the literature. In Australia, potential e-health literacy–related usability issues were found within My Health Record, the national EPHR system. [38]. Moreover, according to Lyles et al., poor health status and limited digital literacy make difficult to access the EPHR [39]. The EPHR system use may present difficulties for several individuals. It is necessary to implement strategies to improve the usability of these systems, particularly for those with limited technology skills or low health or literacy levels [40].

Furthermore, those born abroad have probably not heard of the EPHR, as previously confirmed by another study that showed that geographical origin is a significant factor in EPHR awareness [14]. Given that the foreign population in Italy is about 8.5%, and in Piedmont, more than 9%, it seems relevant that informative campaigns should consider the foreign population to increase the number of citizens who become aware of the EPHR [41, 42].

Perceived health status influences EPHR awareness, since those affected with poor health conditions and clinical chronicity are likelier to have heard of the EPHR. This result highlights a greater engagement with healthcare services for such groups. Accordingly, several studies emphasize the utility of a service like the EPHR for individuals with chronic conditions, for their higher and periodic demand for healthcare assistance resulting in a broad collection of reports and documentation [7, 8, 14, 43]. Appropriate data digitization might facilitate the verification and organization of a large amount of information, prompting decision-making and improving the quality of assistance. Other authors highlighted the relationship between acceptance of the EPHR and the patient’s physical and mental health: multi-morbidity, the number of medication prescriptions, hospitalizations and having a chronic illness induced a positive opinion about the EPHR [44].

A lack of trust in the HCWs impacts the awareness of the EPHR: believing that a GP or a medical specialist has limited information about patients’ health status is associated with a lower awareness of the EPHR. The recent SARS-CoV-2 pandemic decreased the trust in authorities such as journalists, politicians, scientists/researchers, and even HCWs [45]. This lack of trust might reduce the chances of using the EPHR by the general population. Moreover, a multicentre international study showed, in the Italian context, a lack of continuity of care, with scarce interaction between GPs and medical specialists about patients’ health information [46, 47].

The successful use of EPHRs needs multifaceted skills such as knowledge of health topics and e-health technology. Hence, to implement the EPHR system, targeted strategies should be considered for including all population groups who benefit from such systems, improving readability and acceptance. [31]. This aligns with the leading health promotion theories, such as Roger’s theory of the diffusion of innovation [48]. The distribution of innovation in a specific population follows an S-shaped curve, with subjects (called innovators) that adopt a released innovation, and others (“late majority”) that embrace the innovation after a careful cost-benefit analysis. Questions concerning the KS (mean 6.03 out of 9) highlight an intermediate level of knowledge among those aware of the EPHR. Interviewees who have heard of the EPHR showed gaps in understanding its features (such as access methods or who can access it or input data). Hence, practical tools for EPHR functioning should adopt an easily understandable language. Indeed, short and comprehensive explanatory videos were created and uploaded to the YouTube platform, whose access is freely available on some EPHR Italian regional homepages [49].

In this regard, shifting the balance towards the benefits of the EPHR, as opposed to its costs, can be achieved by enhancing its usability, or increasing the system’s user capabilities. This could involve improving digital literacy to enable smoother usage. Additionally, utilizing peer influence as a catalyst for change could also contribute to a broader adoption of the record [7].

The multivariable linear regression model showed that age is the only parameter influencing the KS, with an inverse relationship. This confirms literature data, showing a correlation between age and the percentage of individuals who activate and continue to use the EPHR even after enrollment [14]. A study conducted in Poland highlighted higher interest in accessing the EPHR among young Polish people, with a decreasing trend over the older age groups. [50]. Results from the UK reported that the middle age group was more confident and aware of EPHR access [37]. In this regard, a correlation between age and internet use is notoriously recognized. Therefore, using the EPHR could be encouraged by fostering digital literacy, especially among older people, and a user-friendly interface. Bridging the knowledge and skills gap between those who use the internet fully autonomously and those who lack digital proficiency would enable greater utilization of services and tools such as EPHR and telemedicine [7].

According to the present analysis, attitudes, and knowledge are somehow associated. Both scores reciprocally increase, suggesting that the EPHR’s exhaustive comprehension of functions, aims, and potentialities prompts favourable opinions among users. It also represents an encouraging opportunity for promoting and implementing the EPHR systems among the population. A significant culture change is required to engage and enable patients to use the EPHR, simultaneously inducing social and organizational changes. Despite consumers’ interest and benefits supporting the adoption of the EPHR, its use remains low, defining the EPHR paradox. Conceiving the EPHR as a tool for addressing patients’ needs and preferences and healthcare providers’ involvement and commitment play a crucial role in this regard [15].

Lastly, fair or poor health status correlated negatively with the “attitudes” score towards the EPHR. For those familiar with the EPHR, this might be related to potential service functionality shortcomings, warranting an investigation into identifying and addressing such deficiencies. Conversely, for those unfamiliar with the service, conducting information campaigns becomes even more crucial, as these individuals could benefit the most from the functions of the EPHR.

### Strengths and Limitations of the Study

One of the study’s strengths is the sample size, involving more than 1500 respondents through paper-based and web-based formats for quickly reaching many subjects. In addition, this research is original and innovative, adding relevant information to improve the efficiency and availability of the EPHR service. Sharing the questionnaire on social media platforms such as Facebook allows better management of responses. However, it excludes non-registered or occasional users. Hence, its distribution also occurred in paper form in waiting rooms of local health authorities and vaccination centres to reach those who are less technology-savvy or without any social media profile. Besides the sampling, a high response rate can hardly be achieved in a cross-sectional study based on a survey and gathered data concern a subset of people agreeing with participation. For this reason, the sample lacks representativeness of the population from Piedmont, with a prevalence of women (74.3% vs. 25.7%) and a scarcity of foreign participants (4.41%).

It should also be mentioned that his paper focuses on the EPHR of only one of the twenty-one regions of Italy. The rationale behind this decision is related to the need to focus on the analysis of a specific type of EPHR, in order to compare the answers obtained by persons using the same device. Further studies can be conducted to compare different types of Italian EPHRs [30].

The sample also seems not representative of the Italian and Piedmont population because most respondents were from cities and towns of < 5000 and 5000–30,000 inhabitants. However, the mean inhabitants of the municipalities of Piedmont are around 3600 (median 971 inhabitants), while the mean inhabitants of the whole Italian municipalities are around 15,000 [51].

## Conclusion

The current research raises issues about several aspects of the EPHR and its management. The analysis confirms a lack of awareness regarding the existence of the EPHR, especially among certain disadvantaged demographic groups. The results of the present study should serve as a driving force for a powerful campaign tailored to specific categories of individuals for enhancing knowledge and usage about the EPHR. Furthermore, the study’s findings showed how it is necessary to simplify the entire process of access to the EPHR and make it more user-friendly, especially for people with lower digital literacy. Healthcare professionals’ efforts to enhance and encourage EPHR use by their patients could help in this process.

Since there are no studies regarding the implementation of the EPHR in the other Italian regions, further researchers are required to generalize the assumptions of the results, with the main aim of increasing the use of the EPHR, and subsequently enhancing healthcare quality.

## Electronic Supplementary Material

Below is the link to the electronic supplementary material.

Supplementary Material 1

## Data Availability

The data that support the findings of this study are available on request from the corresponding author, [MM]. The data are not publicly available due to privacy restriction.
