# Supplementary material for: Knowledge, Attitudes, and Practices about Electronic Personal Health Records: A Cross-Sectional Study in a Region of Northern Italy
Source: J Med Syst. 2024 Apr 17;48(1):42. doi: 10.1007/s10916-024-02065-z (PMC11023976; doi:10.1007/s10916-024-02065-z)
Supplement: Supplementary file 1 — Supplementary Material 1 [file 10916_2024_2065_MOESM1_ESM.docx]

| **Table S 1. Knowledge about the EPHR** | | | | |
| --- | --- | --- | --- | --- |
| **Variable** | | | **n** | **%** |
| As you know, is the electronic health record system active in your region of residency? | | Yes | 834 | 86,51% |
|  |  | No | 13 | 1,35% |
|  |  | I don’t know | 117 | 12,14% |
| In your opinion, the function of the electronic health record system is to: | Allowing doctors to access patients' health information, | Yes | 871 | 91,11% |
|  |  | No | 85 | 8,89% |
|  | Enabling the execution of telemedicine appointments | Yes | 276 | 29,24% |
|  |  | No | 668 | 70,76% |
|  | Enabling the government to monitor citizens' income situation, | Yes | 79 | 8,34% |
|  |  | No | 868 | 91,66% |
|  | Facilitating the booking of tests and specialist appointments, | Yes | 755 | 79,39% |
|  |  | No | 196 | 20,61% |
|  | Enabling patients to access their own health information, | Yes | 946 | 98,64% |
|  |  | No | 13 | 1,36% |
|  | Facilitating communication between the General Practitioner (Family Doctor) and Specialist Doctor, | Yes | 674 | 71,47% |
|  |  | No | 269 | 28,53% |
| After activating the Electronic Health Record, how do you access it? | | Through email /PEC | 30 | 3,12% |
|  |  | By visiting your General Practitioner/Local Health Authority | 7 | 0,73% |
|  |  | Through digital identity/Electronic Identity Card/Health Card | 912 | 94,80% |
|  |  | By visiting local pharmacies, | 13 | 1,35% |
| Who can access the Electronic Health Record? | | The citizen only | 114 | 11,84% |
|  |  | The doctors only | 17 | 1,77% |
|  |  | Both the doctors and the citizen, but the citizen decides whether and what information the doctor can access, | 134 | 13,91% |
|  |  | Both the doctors and the citizen, but the doctor decides whether and what information the citizen can access, | 698 | 72,48% |
| Who can enter data into the Electronic Health Record? | | The citizen only | 63 | 6,59% |
|  |  | The doctors/local health authority only | 262 | 27,41% |
|  |  | Both the doctors/local health authority and the patient | 477 | 49,90% |
|  |  | The Regional authority | 154 | 16,11% |

| **Table S2.Attitudes about the EPHR** | | | |
| --- | --- | --- | --- |
| **Variable** | | **n** | **%** |
| The Personal Electronic Health Record is a useful tool for sharing information among different doctors who examine me. | Strongly disagree | 58 | 3,77% |
|  | Disagree | 49 | 3,18% |
|  | Agree | 713 | 46,30% |
|  | Strongly agree | 720 | 46,75% |
| The Electronic Health Record increases my knowledge about my health status. | Strongly disagree | 57 | 3,71% |
|  | Disagree | 155 | 10,08% |
|  | Agree | 834 | 54,23% |
|  | Strongly agree | 492 | 31,99% |
| The Electronic Health Record can ensure a better quality of care. | Strongly disagree | 48 | 3,12% |
|  | Disagree | 140 | 9,09% |
|  | Agree | 841 | 54,61% |
|  | Strongly agree | 511 | 33,18% |
| It is helpful for the General Practitioner (Family Doctor) and other doctors who examine me to have access to information about my previous visits, tests and treatments. | Strongly disagree | 42 | 2,73% |
|  | Disagree | 22 | 1,43% |
|  | Agree | 619 | 40,25% |
|  | Strongly agree | 855 | 55,59% |
| It is helpful for the General Practitioner and other doctors who examine me to be able to exchange information about my previous visits, tests and treatments. | Strongly disagree | 43 | 2,80% |
|  | Disagree | 37 | 2,41% |
|  | Agree | 672 | 43,69% |
|  | Strongly agree | 786 | 51,11% |

| **Table S3. Have you ever heard about EPHR? Socio-demographic variables** | | **No** | | **Yes** | |  |
| --- | --- | --- | --- | --- | --- | --- |
| **Variabile** |  | **n** | **%** | **n** | **%** | **p-value** |
| Gender | Male | 182 | 45.39% | 219 | 54.61% | **0.002** |
|  | Female | 425 | 36.45% | 741 | 63.55% |  |
| Age | Mean and standard deviation | *48.24* | *±15.08* | *48.01* | *±12.80* | 0.674 |
| Nationality | Italian | 560 | 37.36% | 939 | 62.64% | **<0.001** |
|  | Foreigner | 43 | 62.32% | 26 | 37.68% |  |
| Place of residence | ≤ 5,000 inhabitants | 253 | 33.64% | 499 | 66.36% | **<0.001** |
|  | 5,001 - 30,000 inhabitants | 206 | 38.94% | 323 | 61.06% |  |
|  | 30,001 - 50,000 inhabitants | 54 | 50.47% | 53 | 49.53% |  |
|  | 50,001 - 100,000 inhabitants | 51 | 57.30% | 38 | 42.70% |  |
|  | 100,001 - 250,000 inhabitants | 7 | 50% | 7 | 50% |  |
|  | >250,000 inhabitants | 35 | 44.30% | 44 | 55.70% |  |
| Study title | None | 3 | 60% | 2 | 40% | **<0.001** |
|  | Primary school | 10 | 76.92% | 3 | 23.08% |  |
|  | Middle school | 138 | 56.10% | 108 | 43.90% |  |
|  | High School | 308 | 37.98% | 503 | 62.02% |  |
|  | College degree | 123 | 30.75% | 277 | 69.25% |  |
|  | PhD | 25 | 25.77% | 72 | 74.23% |  |
| Profession | Student | 28 | 56% | 22 | 44% | **<0.001** |
|  | Employed | 376 | 34.69% | 708 | 65.31% |  |
|  | Housekeeper | 56 | 55.45% | 45 | 44.55% |  |
|  | Retired | 106 | 43.09% | 140 | 56.91% |  |
|  | Unemployed | 28 | 44.44% | 35 | 55.56% |  |
|  | Other | 13 | 46.43% | 15 | 53.57% |  |
| Healthcare worker | No | 538 | 41.19% | 768 | 58.81% | **<0.001** |
|  | Yes | 68 | 25.76% | 196 | 74.24% |  |
| Lives alone | No | 513 | 37.07% | 871 | 62.93% | **0.001** |
|  | Yes | 93 | 49.73% | 94 | 50.27% |  |

**p-value<0.05** *(bold numbers are significant results)*

| **Table S4. Have you ever heard about EPHR? Health-related variables** | | **No** | | **Yes** | |  |
| --- | --- | --- | --- | --- | --- | --- |
| **Variabile** | | **n** | **%** | **n** | **%** | **p-value** |
| How do you consider your health status? | Excellent | 111 | 36.39% | 194 | 63.61% | 0.227 |
|  | Good | 360 | 40.91% | 520 | 59.09% |  |
|  | Fair | 126 | 35.69% | 227 | 64.31% |  |
|  | Poor | 11 | 32.35% | 23 | 67.65% |  |
| Do you have any chronic illnesses? | No | 343 | 41.83% | 477 | 58.17% | **0.005** |
|  | Yes | 262 | 34.93% | 488 | 65.07% |  |
| How often have you been visited by a doctor / hospitalized in the last three months? | Never | 379 | 42.44% | 514 | 57.56% | **0.001** |
|  | Once | 130 | 34.03% | 252 | 65.97% |  |
|  | Twice | 45 | 31.47% | 98 | 68.53% |  |
|  | Three times | 24 | 38.10% | 39 | 61.90% |  |
|  | More than three times | 30 | 32.97% | 61 | 67.03% |  |
| How often do you have someone helping you read medical materials (such as reports, discharge letters)? | Always | 30 | 48.39% | 32 | 51.61% | **0.016** |
|  | Often | 42 | 45.16% | 51 | 54.84% |  |
|  | Sometimes | 82 | 43.39% | 107 | 56.61% |  |
|  | Rarely | 130 | 41.80% | 181 | 58.20% |  |
|  | Never | 321 | 35.12% | 593 | 64.88% |  |
| How confident do you feel in filling out medical forms on your own? | Very much | 78 | 27.08% | 210 | 72.92% | **<0.001** |
|  | A lot | 167 | 32.75% | 343 | 67.25% |  |
|  | Quite a bit | 274 | 44.26% | 345 | 55.74% |  |
|  | A little | 67 | 51.94% | 62 | 48.06% |  |
|  | Not at all | 23 | 85.19% | 4 | 14.81% |  |
| When you visit a doctor or specialist, do you bring documents concerning your health condition? | Always | 297 | 32.89% | 606 | 67.11% | **<0.001** |
|  | Often | 136 | 38.97% | 213 | 61.03% |  |
|  | Rarely | 117 | 52.00% | 108 | 48.00% |  |
|  | Never | 58 | 61.05% | 37 | 38.95% |  |
| Do you use a computer or smartphone to manage your health? | No | 5 | 55.56% | 4 | 44.44% | 0.320 |
|  | Yes | 604 | 38.59% | 961 | 61.41% |  |
| Do you believe that your General Practitioner (Family Doctor) has the necessary information about your health? | Yes. complete information | 155 | 36.30% | 272 | 63.70% | 0.118 |
|  | Yes. but not complete | 285 | 37.85% | 468 | 62.15% |  |
|  | Yes. a minority of the information | 85 | 37.28% | 143 | 62.72% |  |
|  | Very few/no information | 67 | 47.52% | 74 | 52.48% |  |
| Do you believe that the specialist doctors who have examined you in the past had the necessary information about your health? | Yes. complete information | 62 | 38.04% | 101 | 61.96% | **0.001** |
|  | Yes. but not complete | 223 | 41.92% | 309 | 58.08% |  |
|  | Yes. a minority of the information | 125 | 35.01% | 232 | 64.99% |  |
|  | Very few/no information | 24 | 66.67% | 12 | 33.33% |  |

**p-value<0.05** *(bold numbers are significant results)*

| **Table S5. Multivariable linear regression analysis. Outcome: Knowledge Score (KS).**  **All the variables of the table were included in the multivariable model.** | | | |
| --- | --- | --- | --- |
| **Variabile** | | **Coeff** | **95% CI** |
| Gender | Female | -0.20 | (-0.28); 0.05 |
| Age |  | **-0.01** | **(-0.01) ; (-0.003)** |
| Place of residence | ≤ 5,000 inhabitants | Ref | Ref |
|  | 5,001 - 30,000 inhabitants | 0.03 | (-0.12); 0.18 |
|  | 30,001 - 50,000 inhabitants | 0.20 | (-0.12); -0.52 |
|  | >50,000 inhabitants | 0.09 | (-0.18); 0.36 |
| Study title | Middle school or lower | Ref | Ref |
|  | High School | -0.03 | (-0.26); 0.20 |
|  | College degree or higher | 0.11 | (-0.13); 0.36 |
| Profession | Student/employer | Ref | Ref |
|  | Housekeeper/retired/ unemployed | 0.11 | (-0.09); 0.31 |
| How do you consider your health status? | Excellent/good | Ref | Ref |
|  | Fair/poor | -0.03 | (-0.20); 0.13 |
| How often do you have someone helping you read medical materials (such as reports, discharge letters)? | Always | Ref | Ref |
|  | Often | **0.80** | **0.31-1.29** |
|  | Sometimes | **0.64** | **0.19; 1.08** |
|  | Rarely | **0.79** | **0.37; -1.21** |
|  | Never | **0.78** | **0.38; 1.18** |
| How confident do you feel in filling out medical forms on your own? | Very much | Ref | Ref |
|  | A lot | -0.04 | (-0.23); 0.15 |
|  | Quite a bit | -0.15 | (-0.35); 0.04 |
|  | A little | -0.09 | (-0.40); 0.23 |
|  | Not at all | -0.98 | (-2.39); 0.42 |
| Filled a questionnaire in paper |  | **0.46** | **0.07; 0.84** |

**p-value<0.05** *(bold numbers are significant results)*

Abbreviation: Coeff. – coefficient; Ref. – Reference value; CI – Confidence Interval

| **Table S6. Multivariable linear regression analysis. Outcome: Attitude Score (AS).**  **All the variables of the table were included in the multivariable model.** | | | |
| --- | --- | --- | --- |
| **Variabile** | | **Coeff** | **95% CI** |
| Gender | Female | -0.42 | (-0.87); 0.03 |
| Age |  | **-0.03** | **(-0.05) ; (-0.02)** |
| Study title | Middle school or lower | Ref | Ref |
|  | High School | -0.23 | (-0.40); 0.85 |
|  | College degree or higher | 0.20 | (-0.48); 0.87 |
| Profession | Student/employer | Ref | Ref |
|  | Housekeeper/retired/ unemployed | -0.05 | (-0.58); 0.49 |
| Health care worker | Yes | -0.06 | (-0.53); 0.42 |
| Lives alone | Yes | 0.11 | (-0.52); 0.74 |
| Do you have any chronic illnesses? | Yes | -0.28 | (-0.66); 0.10 |
| How confident do you feel in filling out medical forms on your own? | Very much | Ref | Ref |
|  | A lot | -0.004 | (-0.52); 0.51 |
|  | Quite a bit | -0.49 | (-1.01); 0.03 |
|  | A little | -0.41 | (-1.24); 0.42 |
|  | Not at all | 2.34 | (-1.63); 6.31 |
| Do you believe that your General Practitioner (Family Doctor) has the necessary information about your health? | Yes. complete information | Ref |  |
|  | Yes. but not complete | -0.12 | (-0.59); 0.34 |
|  | Yes. a minority of the information | -0.51 | (-1.14); 0.12 |
|  | Very few/no information | -0.89 | (-1.69); (-0.09) |
| Do you believe that the specialist doctors who have examined you in the past had the necessary information about your health? | Yes. complete information | Ref |  |
|  | Yes. but not complete | 0.31 | (-0.39); 0.99 |
|  | Yes. a minority of the information | 0.62 | (-0.11); 1.36 |
|  | Very few/no information | 0.46 | (-0.24); 1.17 |
|  | A Specialist has never examined me. | -1.11 | (-2.95); 0.71 |
| Knowledge Score KS |  | **0.27** | **0.09; 0.44** |

**p-value<0.05** *(bold numbers are significant results)*

Abbreviation: Coeff. – coefficient; Ref. – Reference value; CI – Confidence Interval
